# A Fast and in-Situ Measuring Method Using Laser Triangulation Sensors for the Parameters of the Connecting Rod

**DOI:** 10.3390/s16101679

**Published:** 2016-10-12

**Authors:** Xing-Qiang Li, Zhong Wang, Lu-Hua Fu

**Affiliations:** State Key Laboratory of Precision Measuring Technology and Instruments, Tianjin University, Tianjin 300072, China; lxq.792751045@163.com (X.-Q.L.); wangzhong@tju.edu.cn (Z.W.)

**Keywords:** in-situ measurement, laser triangulation sensor, the connecting rod

## Abstract

The connecting rod is a critical part inside the marine engine. The inspection of its important parameters is directly related to the assembly and quality of the marine engine. A coordinate measuring machine (CMM) is a conventional choice to measure the parameters of a connecting rod. However, CMM requires significant resources in time and cost, which leads researchers into in-situ measurement. This article presents a fast and in-situ measuring method by using a laser-based measuring head. Two measuring strategies are adopted in the inspection process. For positional measurements (such as the hole–center distance), whose accuracy requirement is generally low, the coordinate system of the numerical control (NC) machine is combined with the measuring head to acquire the positional parameters. For dimensional measurements (such as inner diameters), whose accuracy requirement is rather high, the NC machine is used just as transportation. Note that the measuring head has the ability to perform the dimension inspection independently. The accuracy of the measuring head is high enough to meet the dimensional accuracy requirements. Experiments are performed to validate the proposed method. The measuring error of the inner diameters is from 5 μm to 7 μm. The measuring error of hole–center distance is within 15 μm. The measurement of all these parameters can be done within 1 min.

## 1. Introduction

Two processes are generally involved when manufacturing a workpiece. One is the machining process, which is carried out to machine the parts; the other is the inspection process, which is performed to check whether the part is machined according to the requirements. For the fabrication of marine engines, an inspection process usually takes more time than a machining process. Therefore, it is necessary to develop a fast measuring method to improve the time efficiency.

A coordinate measuring machine (CMM) is a general choice for dimension inspections due to its accuracy and flexibility [1]. In the case of the fabrication of marine engines, however, the time cost generated by transferring these large workpieces onto a CMM is the primary barrier to this method [2,3]. In addition, given this consideration, in-situ measurement seems a promising solution that is able to machine and measure simultaneously [4]. Inspired by CMM, some companies [5,6] embedded trigger probes [7] into numerical control (NC) machines for in-situ measurement. Nevertheless, the trigger probes are easily broken and large-scale machine tools are hard to control subtly. Non-contact measuring methods have also drawn much attention because they are fast and much safer than contact methods. Ito et al. [8] used a laser displacement sensor (LDS) to measure the surface form of ceramics parts. Due to the limitation of its accuracy, however, this kind of method is commonly used for form measurement but not dimension inspection, such as in [9,10,11,12]. The main reason is understandable as the coordinates offered by the NC machines are less accurate, and thus the final measuring accuracy is not comparable to that of the CMM yet.

Research has been carried out on the error compensation of NC machines. Ibaraki et al. [13] formulated the geometric errors of machines by using a LDS. Similar research achievements have also been published in [14,15,16]. Hong et al. [17] developed a non-contact R-test device for calibrating the NC machines. Janusiewicz et al. [18] analyzed the impact of the theoretical error when performing an in-situ roundness measurement. The aforementioned methods offer a possible direction for precise in-situ measurement, but most compensation methods are time-consuming and need to be executed each time before measurements. These drawbacks become an obstacle to practical use.

**Figure 1:**
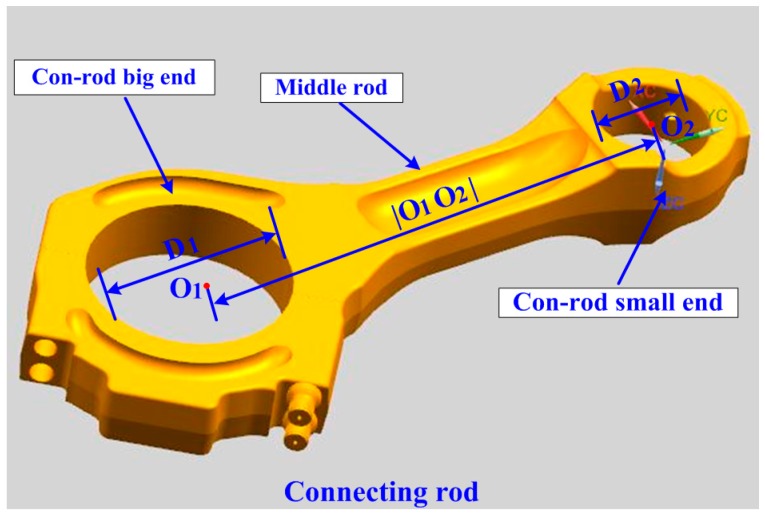
The connecting rod.

The connecting rod is a critical part inside the marine engine [19]. As shown in Figure 1, a connecting rod is composed of three portions: the con-rod big end, the middle rod, and the con-rod small end. The machining quality of a connecting rod is directly related to the assembly of the marine engine [20]. Hence, the crucial parameters of the connecting rod, which determine its final quality, should be inspected seriously before assembly. By using a laser-based measuring head, a fast and in-situ measuring method is proposed in this article for the measuring the parameters of the connecting rod, including the two inner diameters of the con-rod ends (*D*_1_, *D*_2_) and the hole–center distance (|O_1_O_2_|). The main contributions in this paper include:

(1) Different from the probes in [5,6], the developed measuring head is able to measure inner diameters independently. Its installation position and the movement accuracy of the machines have no effect on the measurement results.
(2) The measuring head can also be used to measure the position parameters of the connecting rod with the help of the NC system. Compared with such measuring heads in [5,6,12], the developed head does not need on-machine calibration. In brief, the head supports “plug-and-measure”.
(3) The data processing system is free from the NC system. Hence, it is easy to integrate the measuring head into diverse NC systems, regardless of the compatibility problem.
(4) The measuring data are transmitted through wireless communication. In addition, this makes it possible to realize remote control.

This article is organized as follows. The measuring principle is introduced in Section 2. In Section 3, the mathematical measurement models are described in detail. The error discussion on practical applications is analyzed in Section 4. The proposed method is validated by experiments in Section 5. The conclusion is finally given in Section 6.

## 2. The Measuring Principle

**Figure 2:**
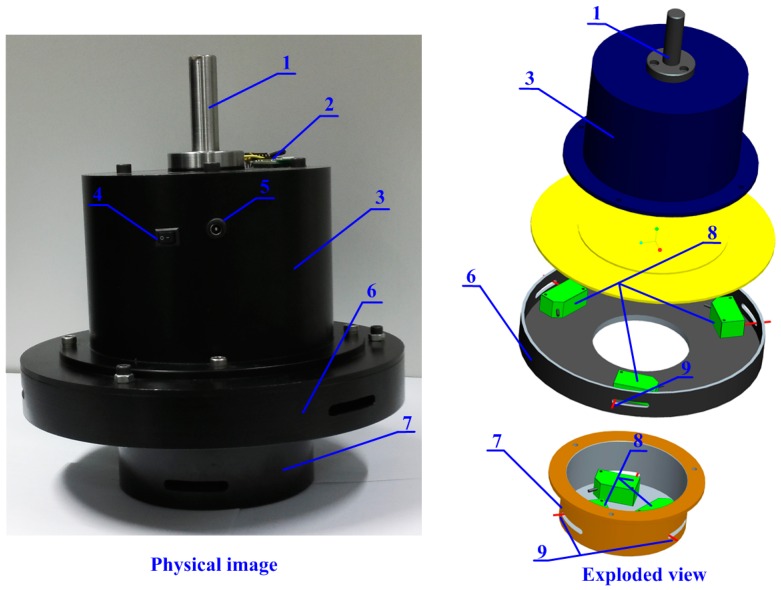
The laser-based measuring head. 1—joint rod, 2—Bluetooth, 3—data processing module, 4—power switch, 5—charging interface, 6—upper bore gauge, 7—lower bore gauge, 8—LDSs, 9—measuring lasers.

In this article, a laser-based measuring head is developed to measure these crucial parameters. The structure of the measuring head is shown in Figure 2. The joint rod is used to install it on NC machines. The LDSs are mounted in the internal circumference of the bore gauges at equal intervals. Data samples can be obtained by these LDSs. The lower bore gauge is used to measure the con-rod small end whereas the upper bore gauge is used to measure the con-rod big end. The sizes of the bore gauges are designed in accordance with the measured holes. The measuring information is preliminarily organized by the data processing module, and then transmitted to the upper computer through Bluetooth.

Two measuring strategies are adopted according to diverse accuracy requirements. For position parameters, whose accuracy requirement is generally low, the position accuracy of NC machines is high enough to deal with this situation [21]. Hence, the coordinate system of NC machines can be employed to measure the position parameters. For dimension parameters, the machining requirement is usually extremely strict. In this case, NC machines are used just for delivery and transportation, and their coordinates have no impact on the measurement results. Note that the developed measuring head is capable of measuring inner diameters independently. Its measuring accuracy can sufficiently meet the exact demand.

In the following section, the measurement model of inner diameters and the hole–center distance are described in detail.

## 3. The Mathematical Measurement Model

### 3.1. Measuring of Inner Diameter

**Figure 3:**
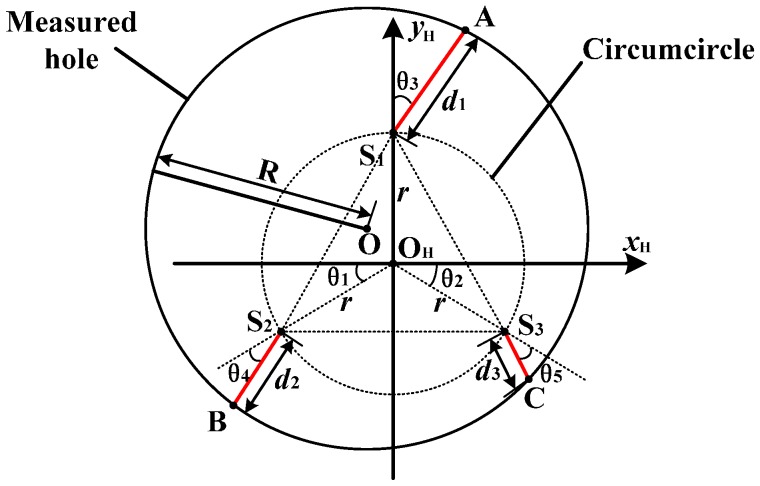
The measurement model of the inner diameter.

The measurement model of the inner diameter is shown in Figure 3. O is the center of the measured hole. S_1_, S_2_, S_3_ are the emitting points of the measuring lasers. A, B, C are the measured points. To establish the relationship between the measured diameter (assumed as *R*) and the measuring head, the coordinate system *x*_H_-*o*_H_-*y*_H_ is introduced, where O_H_ is the center of the circumcircle of triangle S_1_S_2_S_3_ and S_1_ is on the positive *y*_H_ axis. Then, A(*x*_1_, *y*_1_), B(*x*_2_, *y*_2_), C(*x*_3_, *y*_3_) can be expressed as:

(1) $$\{\begin{array}{l} x_{1}=d_{1}\cdot \sin\theta_{3}\\ y_{1}=r+d_{1}\cdot \cos\theta_{3} \end{array}\{\begin{array}{l} x_{2}=-[r\cdot \cos\theta_{1}+d_{2}\cdot \cos(\theta_{1}+\theta_{4})]\\ y_{2}=-[r\cdot \sin\theta_{1}+d_{2}\cdot \sin(\theta_{1}+\theta_{4})] \end{array}\{\begin{array}{l} x_{3}=r\cdot \cos\theta_{2}+d_{3}\cdot \cos(\theta_{2}+\theta_{5})\\ y_{3}=-[r\cdot \sin\theta_{2}+d_{3}\cdot \sin(\theta_{2}+\theta_{5})] \end{array}$$

where *θ*_1_ is the angle between the negative *x*_H_ axis and O_H_S_2_; *θ*_2_ is the angle between the positive *x*_H_ axis and O_H_S_3_; *θ*_3_ is the angle between the positive *y*_H_ axis and S_1_A (clockwise rotation is positive); *θ*_4_ is the angle between O_H_S_2_ and S_2_B (clockwise rotation is negative); *θ*_5_ is the angle between O_H_S_3_ and S_3_C (clockwise rotation is positive); *r* denotes the radius value of the circumcircle; *d*_1_ denotes the value of |S_1_A|; *d*_2_ denotes the value of |S_2_B|; *d*_3_ denotes the value of |S_3_C|. To better describe this measurement model, {*θ*_1_, *θ*_2_, *θ*_3_, *θ*_4_, *θ*_5_, *r*} are defined as the intrinsic parameters of the measuring head. Based on Equation (1), the sides of triangle ABC can be written as:

(2) $$\{\begin{array}{l} \begin{array}{l} a=\left|BC\right|=\sqrt{{\left(x_{2}-x_{3}\right)}^{2}+{\left(y_{2}-y_{3}\right)}^{2}}\\ =\sqrt{{\left[r\cdot \cos\theta_{1}+d_{2}\cdot \cos(\theta_{1}+\theta_{4})+r\cdot \cos\theta_{2}+d_{3}\cdot \cos(\theta_{2}+\theta_{5})\right]}^{2}+{\left[r\cdot \sin\theta_{1}+d_{2}\cdot \sin(\theta_{1}+\theta_{4})-r\cdot \sin\theta_{2}-d_{3}\cdot \sin(\theta_{2}+\theta_{5})\right]}^{2}} \end{array}\\ \begin{array}{l} b=\left|AC\right|=\sqrt{{\left(x_{1}-x_{3}\right)}^{2}+{\left(y_{1}-y_{3}\right)}^{2}}\\ =\sqrt{{\left[d_{1}\cdot \sin\theta_{3}-r\cdot \cos\theta_{2}-d_{3}\cdot \cos(\theta_{2}+\theta_{5})\right]}^{2}+{\left[r+d_{1}\cdot \cos\theta_{3}+r\cdot \sin\theta_{2}+d_{3}\cdot \sin(\theta_{2}+\theta_{5})\right]}^{2}} \end{array}\\ \begin{array}{l} c=\left|AB\right|=\sqrt{{\left(x_{1}-x_{2}\right)}^{2}+{\left(y_{1}-y_{2}\right)}^{2}}\\ =\sqrt{{\left[d_{1}\cdot \sin\theta_{3}+r\cdot \cos\theta_{1}+d_{2}\cdot \cos(\theta_{1}+\theta_{4})\right]}^{2}+{\left[r+d_{1}\cdot \cos\theta_{3}+r\cdot \sin\theta_{1}+d_{2}\cdot \sin(\theta_{1}+\theta_{4})\right]}^{2}} \end{array} \end{array}$$

As shown in Figure 3, the measured hole is the circumcircle of triangle ABC. Hence, the measured diameter can be expressed as below:

(3) $$R=\frac{a\cdot b\cdot c}{\sqrt{\left(a+b+c)\cdot (a+b-c)\cdot (a+c-b)\cdot (b+c-a\right)}}$$

By substituting Equation (2) into Equation (3), Equation (4) can be deduced.

(4) $$R=f_{1}(\theta_{1},\theta_{2},\theta_{3},\theta_{4},\theta_{5},r,d_{1,i},d_{2,i},d_{3,i})$$

where *i* is the group number of measurements; *f*_1_(∙) is defined as the function to express the relationship between the measured diameter and the intrinsic parameters. In addition, the center of the measured hole (labeled as point O in Figure 3) can be written as:

(5) $$\{\begin{array}{c} x_{0}^{H}=\frac{\left(x_{1}^{2}-x_{2}^{2}+y_{1}^{2}-y_{2}^{2})\cdot (y_{1}-y_{3})-(x_{1}^{2}-x_{3}^{2}+y_{1}^{2}-y_{3}^{2})\cdot (y_{1}-y_{2}\right)}{2\cdot (y_{1}-y_{3})\cdot (x_{1}-x_{2})-2\cdot (y_{1}-y_{2})\cdot (x_{1}-x_{3})}\\ y_{0}^{H}=\frac{\left(x_{1}^{2}-x_{2}^{2}+y_{1}^{2}-y_{2}^{2})(x_{1}-x_{3})-(x_{1}^{2}-x_{3}^{2}+y_{1}^{2}-y_{3}^{2})(x_{1}-x_{2}\right)}{2\cdot (y_{1}-y_{2})(x_{1}-x_{3})-2\cdot (y_{1}-y_{3})(x_{1}-x_{2})} \end{array}$$

where ( $x_{0}^{H}$ , $y_{0}^{H}$ ) represents the coordinates of point O. By inserting Equation (1) into Equation (5), Equation (6) can be deduced:

(6) $$\{\begin{array}{c} x_{0}^{H}=f_{2}(\theta_{1},\theta_{2},\theta_{3},\theta_{4},\theta_{5},r,d_{1,i},d_{2,i},d_{3,i})\\ y_{0}^{H}=f_{3}(\theta_{1},\theta_{2},\theta_{3},\theta_{4},\theta_{5},r,d_{1,i},d_{2,i},d_{3,i}) \end{array}$$

Furthermore, |OO_H_| can be calculated from Equation (7).

(7) $$\left|\text{O}O_{H}\right|=\sqrt{{\left(x_{0}^{H}\right)}^{2}+{\left(y_{0}^{H}\right)}^{2}}=\sqrt{{\left[f_{2}(\cdot )\right]}^{2}+{\left[f_{3}(\cdot )\right]}^{2}}=f_{4}(\theta_{1},\theta_{2},\theta_{3},\theta_{4},\theta_{5},r,d_{1,i},d_{2,i},d_{3,i})$$

where *f*_4_(∙) is defined as the function to express the distance between point O and point O_H_.

Consequently, the diameter of the measured hole can be calculated from Equation (4). Given several groups of measurements ({*d*_1*i*_, *d*_2*i*_, *d*_3*i*_}), the intrinsic parameters can also be calibrated through Equation (4). The contribution of this measurement model is that the inner diameter can be immediately obtained as soon as the measuring head is put inside the measured hole. The movement mechanism of NC machines has nothing to do with the measurement results.

### 3.2. Measuring of Hole–Center Distance

**Figure 4:**
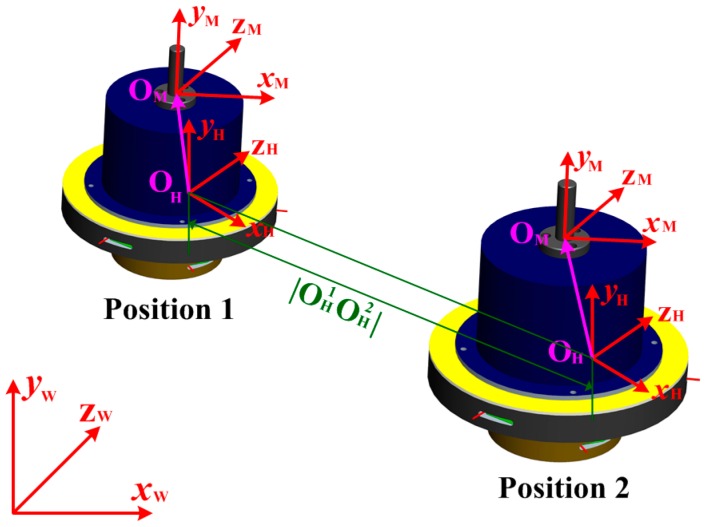
Coordinate system transformation.

As analyzed above, the position relationship between the measuring head and the hole–center distance can be obtained by Equation (7). To measure the hole–center distance, generally speaking, the relative position between the measuring head and the NC machine tools should also be formulated. To clarify this, the coordinate system *x*_M_-*y*_M_-*z*_M_ is introduced as the coordinate system of NC machines (CSNC). The coordinate system *x*_H_-*y*_H_-*z*_H_ is defined as the coordinate system of the measuring head (CSMH). Then *x*_W_-*y*_W_-*z*_W_ is the world coordinate system (WCS). As shown in Figure 4, when the measuring head is moved by the motion mechanism of NC machines, the relationship between CSNC and CSMH remains the same. Assume that

(8) $$\vec{O_{H}O_{M}}=(x_{M}-x_{H},y_{M}-y_{H},z_{M}-z_{H})=(l,m,n)$$

where (*x*_M_, *y*_M_, *z*_M_) denotes the spindle coordinate in the CSNC; (*x*_H_, *y*_H_, *z*_H_) represents the origin coordinate of the CSMH in the WCS. Once the measuring head is installed onto the spindle of NC machines, $\vec{O_{H}O_{M}}$ will be the inherent parameter in the measuring system and will not change.

If the measuring head moves from Position 1 to Position 2, the moving distance can be expressed as:

(9) $$\left|O_{H}^{1}O_{H}^{2}\right|=\sqrt{{\left(x_{H}^{1}-x_{H}^{2}\right)}^{2}+{\left(y_{H}^{1}-y_{H}^{2}\right)}^{2}+{\left(z_{H}^{1}-z_{H}^{2}\right)}^{2}}$$

where ( $x_{H}^{1}$ , $y_{H}^{1}$ , $z_{H}^{1}$ ) represents the origin coordinate in the WCS at Position 1; ( $x_{H}^{2}$ , $y_{H}^{2}$ , $z_{H}^{2}$ ) denotes the origin coordinate in the WCS at Position 2. By inserting Equation (8) into Equation (9), Equation (10) can be deduced:

(10) $$\begin{array}{ll} \left|O_{H}^{1}O_{H}^{2}\right| & =\sqrt{{\left(x_{H}^{1}-x_{H}^{2}\right)}^{2}+(y_{H}^{1}-y_{H}^{2}{)}^{2}+{\left(z_{H}^{1}-z_{H}^{2}\right)}^{2}}\\ & =\sqrt{{\left[(x_{M}^{1}-l)-(x_{M}^{2}-l)\right]}^{2}+{\left[(y_{M}^{1}-m)-(y_{M}^{2}-m)\right]}^{2}+{\left[(z_{M}^{1}-n)-(z_{M}^{2}-n)\right]}^{2}}\\ & =\sqrt{{\left(x_{M}^{1}-x_{M}^{2}\right)}^{2}+{\left(y_{M}^{1}-y_{M}^{2}\right)}^{2}+{\left(z_{M}^{1}-z_{M}^{2}\right)}^{2}} \end{array}$$

where ( $x_{M}^{1}$ , $y_{M}^{1}$ , $z_{M}^{1}$ ) is the spindle coordinate in the CSNC at Position 1; ( $x_{M}^{2}$ , $y_{M}^{2}$ , $z_{M}^{2}$ ) is the spindle coordinate in the CSNC at Position 2. Note that the spindle coordinates of the CSNC can be directly read from the NC system. The moving distance of the measuring head can be calculated from Equation (10).

From Equations (7) and (10), we can come up with two important deductions:

Deduction 1 The distance between the measuring head and measured hole–center distance can be calculated from Equation (7).
Deduction 2 When the measuring head moves from Position 1 to Position 2, its moving distance can be acquired by the coordinate change of the CSNC.

**Figure 5:**
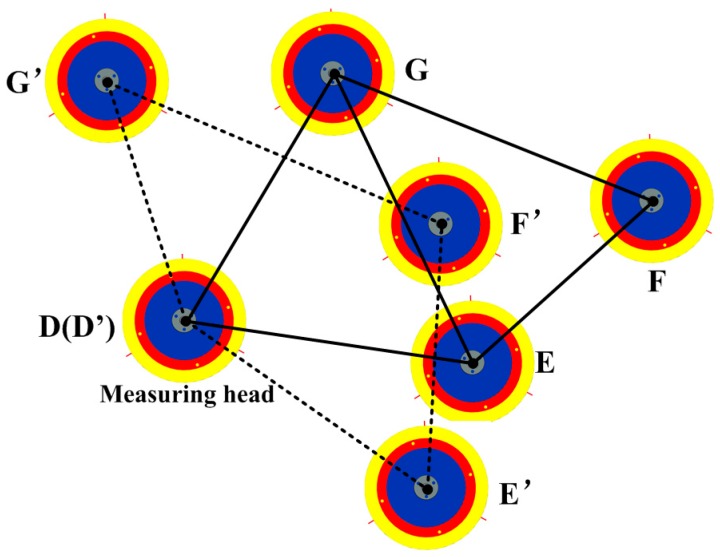
The measurement model of double triangles.

The deductions above offer some necessary information about measuring distances. Nevertheless, no deductions about measuring angles have been obtained yet. As a result, it is difficult to calculate the hole–center distance simply by the two deductions above. For example, as shown in Figure 5, the shape of quadrangle DEFG is not fixed if we merely give the lengths of its four sides (|*DE*|, |*EF*|, |*FG*|, and |*GD*|). However, the quadrangle’s shape will be fixed under an extra condition that the length of |*EG*| is given. Under this condition, |*DF*| can be calculated by

(11) $$\left|DF\right|=\sqrt{{\left|DG\right|}^{2}+{\left|FG\right|}^{2}-2\cdot \left|DG\right|\cdot \left|FG\right|\cdot \cos∠DGF}$$

where

(12) $$\{\begin{array}{l} ∠DGF=∠DGE+∠EGF\\ ∠DGE=\arccos\frac{{\left|DG\right|}^{2}+{\left|EG\right|}^{2}-{\left|DE\right|}^{2}}{2\cdot \left|DG\right|\cdot \left|EG\right|}\\ ∠EGF=\arccos\frac{{\left|EG\right|}^{2}+{\left|FG\right|}^{2}-{\left|EF\right|}^{2}}{2\cdot \left|EG\right|\cdot \left|FG\right|} \end{array}$$

From Equations (11) and (12), we can come up with another deduction:

Deduction 3 If two triangles have a common side and all the sides’ values are given, the distance between the two non-adjacent vertexes is unique and can be calculated by Equations (11) and (12).

In what follows, the hole–center distance will be calculated by these three deductions.

**Figure 6:**
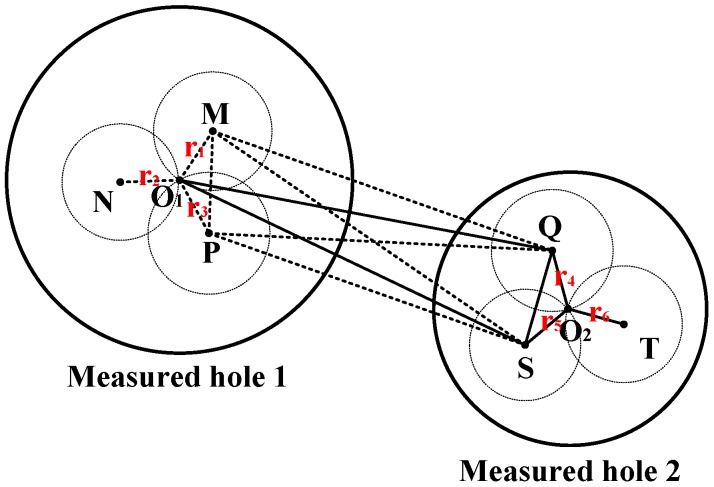
The measurement model of the hole–center distance.

As shown in Figure 6, *O*_1_ is the center of measured hole 1 whereas *O*_2_ is the center of measured hole 2. When the measuring head is put at position M, |*O*_1_*M*| can be obtained by Deduction 1. The radius of circle M is |*O*_1_*M*|. In the nature of things, *O*_1_ should be on circle *M*. In a similar way, *O*_1_ should also be on circle N and circle P, whose radiuses are, respectively, |*O*_1_*N*| and |*O*_1_*P*|. As a result, *O*_1_ can be determined by the unique common point of circle M, circle N, and circle P. Likewise, *O*_2_ can also be fixed by circle Q, circle S, and circle T. Triangle *O*_1_*MP* and triangle *QMP* have a common side, *MP*. Based on Deduction 3, |*O*_1_*Q*| can be calculated by Equation (13).

(13) $$\left|O_{1}Q\right|=\sqrt{{\left|O_{1}M\right|}^{2}+{\left|MQ\right|}^{2}-2\cdot \left|O_{1}M\right|\cdot \left|MQ\right|\cdot \cos∠O_{1}MQ}$$

where

(14) $$\{\begin{array}{l} \begin{array}{l} ∠O_{1}MQ=∠O_{1}MP+∠PMQ \end{array}\\ ∠O_{1}MP=\arccos\frac{{\left|O_{1}M\right|}^{2}+{\left|MP\right|}^{2}-{\left|O_{1}P\right|}^{2}}{2\cdot \left|O_{1}M\right|\cdot \left|MP\right|}\\ ∠PMQ=\arccos\frac{{\left|MP\right|}^{2}+{\left|MQ\right|}^{2}-{\left|PQ\right|}^{2}}{2\cdot \left|MP\right|\cdot \left|MQ\right|} \end{array}$$

Triangle *O*_1_*MP* and triangle *MPS* have a common side *MP*. Based on Deduction 3, |*O*_1_*S*| can be acquired by

(15) $$\left|O_{1}S\right|=\sqrt{{\left|O_{1}M\right|}^{2}+{\left|MS\right|}^{2}-2\cdot \left|O_{1}M\right|\cdot \left|MS\right|\cdot \cos∠O_{1}MS}$$

where

(16) $$\{\begin{array}{l} ∠O_{1}MS=∠O_{1}MP+∠PMS\\ ∠O_{1}MP=\arccos\frac{{\left|O_{1}M\right|}^{2}+{\left|MP\right|}^{2}-{\left|O_{1}P\right|}^{2}}{2\cdot \left|O_{1}M\right|\cdot \left|MP\right|}\\ ∠PMS=\arccos\frac{{\left|MP\right|}^{2}+{\left|MS\right|}^{2}-{\left|PS\right|}^{2}}{2\cdot \left|MP\right|\cdot \left|MS\right|} \end{array}$$

In a similar way, |*O*_1_*O*_2_| can be deduced between triangle *QO*_1_*S* and triangle *QO*_2_*S*.

(17) $$\left|O_{1}O_{2}\right|=\sqrt{{\left|O_{1}Q\right|}^{2}+{\left|QO_{2}\right|}^{2}-2\cdot \left|O_{1}Q\right|\cdot \left|QO_{2}\right|\cdot ∠O_{1}QO_{2}}$$

where

(18) $$\{\begin{array}{l} ∠O_{1}QO_{2}=∠O_{1}QS+∠SQO_{2}\\ ∠O_{1}QS=\arccos\frac{{\left|O_{1}Q\right|}^{2}+{\left|QS\right|}^{2}-{\left|O_{1}S\right|}^{2}}{2\cdot \left|O_{1}Q\right|\cdot \left|QS\right|}\\ ∠SQO_{2}=\arccos\frac{{\left|SQ\right|}^{2}+{\left|QO_{2}\right|}^{2}-{\left|SO_{2}\right|}^{2}}{2\cdot \left|SQ\right|\cdot \left|QO_{2}\right|} \end{array}$$

Based on Deduction 1, |*O*_1_*M*|, |*O*_1_*N*|, |*O*_1_*P*|, |*O*_2_*Q*|, |*O*_2_*S*| and |*O*_2_*T*| are known quantities. Based on Deduction 2, |*MP*|, |*MQ*|, |*PQ*|, |*MS*|, |*PS*|, and |*QS*| are also known quantities. As a result, the hole–center distance |*O*_1_*O*_2_| can be calculated from Equation (17). The contribution of this measurement model is that the measuring head does not need in-situ calibration and the inspection process can be immediately carried out as long as the head is installed onto the NC machines.

## 4. The Error Discussion on Practical Application

### 4.1. Data Sample for Calibrating Intrinsic Parameters

**Figure 7:**
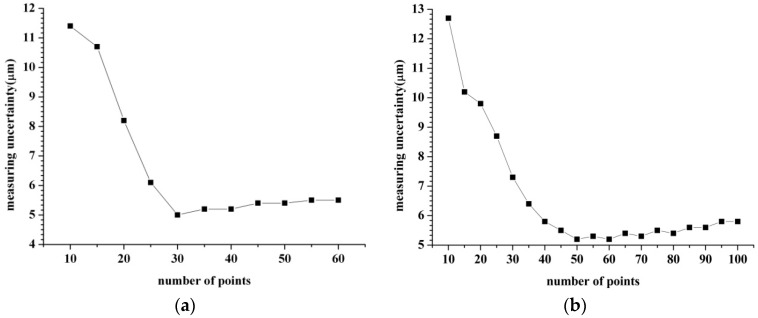
The number selection of calibration points. (**a**) The diameter of the measured hole is 150 mm; (**b**) The diameter of the measured hole is 275 mm.

As in the analysis in Section 3.1, the intrinsic parameters can be calibrated by Equation (4). As the number of the intrinsic parameters is six, we need at least six groups of measurements ({*d*_1*i*_, *d*_2*i*_, *d*_3*i*_}). By changing the relative position between the measuring head and measured hole, plenty of groups of measurements ({*d*_1*i*_, *d*_2*i*_, *d*_3*i*_}) can be acquired. To clarify the influence of the group number *i* on the measurement results, the simulation is performed as shown in Figure 7. In the simulation analysis, the errors of the measurements ({*d*_1*i*_, *d*_2*i*_, *d*_3*i*_}) are assumed as 0.1%. The relative positions are chosen randomly. Two inner diameters of 150 mm and 275 mm are taken as examples. From Figure 7 we can see that, the measurement uncertainty firstly decreases as the number of points increases. The reason for this is that the measurement accuracy improves with increasing the measurement information. Then, the measurement uncertainty increases as the number of points sequentially increases. The reason is understandable as the errors of measurements ({*d*_1*i*_, *d*_2*i*_, *d*_3*i*_}) reduce the final accuracy. In Figure 7a, the measurement uncertainty reaches the minimum value when *i* is approximately 30. In Figure 7b, the measurement result contains the least error when *i* is about 50. From the simulation we can know that *i* should be chosen as 30 while measuring the 150 mm diameter whereas *i* should be given as 50 when measuring the 275 mm diameter.

### 4.2. Actual Measurement of a Connecting Rod

**Figure 8:**
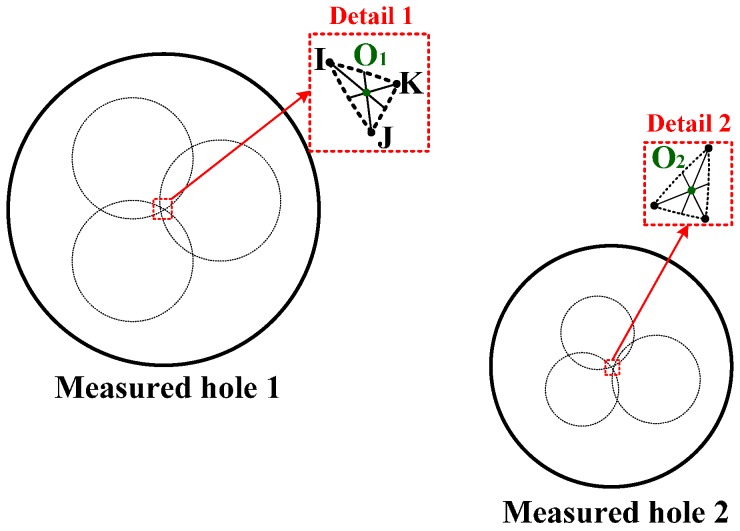
The actual measurement of the connecting rod.

As discussed in Section 3.2, the measured hole–center distance is the common point of the three circles. In practice, the three circles may not have any common point because of the measurement errors of the head and the coordinate errors caused by NC machines. As shown in Figure 8, the common point in theory may be converted into the crossed regions as shown by Details 1 and 2. Since the crossed regions are really small, their shapes can be deemed as triangles. In this case, the measured hole–center distance is regarded as the barycentric coordinate of the triangle. For example, the barycenter of triangle IJK in Detail 1 can be expressed as:

(19) $$\{\begin{array}{l} x_{O1}=\frac{x_{I}+x_{J}+x_{K}}{3}\\ y_{O1}=\frac{y_{I}+y_{J}+y_{K}}{3}\\ z_{O1}=\frac{z_{I}+z_{J}+z_{K}}{3} \end{array}$$

where (*x_O_*_1_, *y_O_*_1_, *z_O_*_1_) is the coordinate of O_1_; (*x*_I_, *y*_I_, *z*_I_) denotes the coordinate of point I; (*x*_J_, *y*_J_, *z*_J_) represents the coordinate of point J; (*x*_K_, *y*_K_, *z*_K_) is defined as the coordinate of K.

## 5. The Experiment

**Figure 9:**
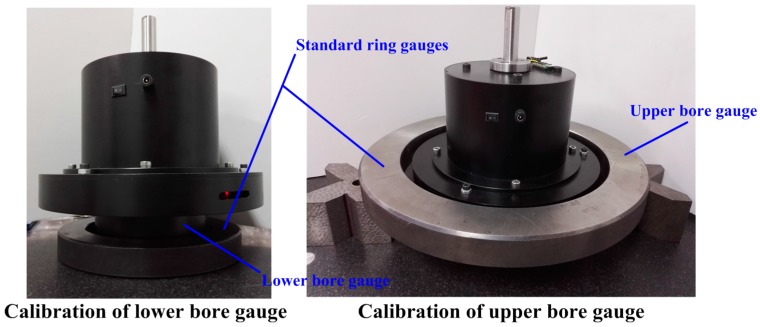
The calibration of the intrinsic parameters.

As shown in Figure 9, the intrinsic parameters are calibrated by using standard ring gauges. The inner diameter of the small ring gauge is 150 mm and the inner diameter of the big ring gauge is 275 mm. The data samples for calibrating the lower bore gauge are collected at 30 measuring positions. The data samples for calibrating the upper bore gauge are gathered at 50 measuring positions. The intrinsic parameters (the definition was given behind Equation (1)) are finally shown in Table 1. The estimated values of the intrinsic parameters can be calculated through the JADE algorithm [22]. The JADE algorithm, proposed by Zhang and Sanderson, is successfully applied to solve numerous optimization problems in diverse fields. It offers great flexibility, robustness and precision with respect to various types of functions. JADE is a mature algorithm and its application detail can refer to [22].

**Figure 10:**
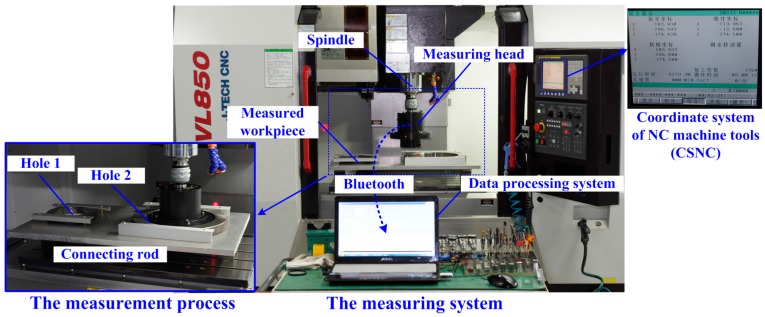
The in-situ measurement system.

The in-situ measurement of a connecting rod is shown in Figure 10. The measurement procedure is described as below:

Step 1 The measuring head is installed onto the spindle of the NC machine.
Step 2 The lower bore gauge of the measuring head is put inside hole 1 (the smaller hole). The measurement data are transmitted to the data processing system through Bluetooth.
Step 3 The relative positions between the measuring head and the measured hole are changed three times and the measurement data with their corresponding coordinates in the CSNC are recorded.
Step 4 In a similar way, the bigger hole can be measured by the upper bore gauge of the measuring head.
Step 5 The measuring data are finally computed by the data processing system and shown on the liquid-crystal display.

The measuring results are show in Table 2. The value of the measurement error can be calculated by the following equation:

Measuring error = Standard value − Measured value(20)

The standard values are provided by a high-accuracy CMM. The verification experiments for the proposed method show that the measurement errors of the dimension parameters are within 10 μm and the measurement error of the position parameter is within 15 μm. Consequently, the proposed method can meet the requirements of the assembly of marine engines. The measurements for these parameters can be done within 1 min.

**Figure 11:**
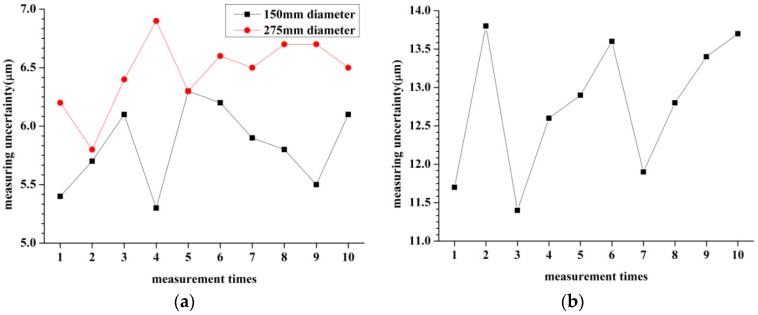
The influence of the installation pose to the measurement results. (**a**) The influence of the installation pose when measuring inner diameters; (**b**) The influence of the installation pose when measuring the hole–center distance.

Note that each time when the measuring head is installed onto the spindle of NC machines, the installation pose cannot be the same. To test the influence of the head’s installation on the measurement results, the head is installed and uninstalled to measure the critical parameters 10 times. The measurement results are shown in Figure 11. From Figure 11a, it can be seen that the measurement uncertainties are all within 10 μm. These data show that the installation parameters do not have obvious effects on the measurements of the inner diameters. It should also be found from Figure 11b that the installation pose does not have a clear relationship with the measurement accuracy.

## 6. Conclusions

A fast and in-situ measuring method is presented in this paper for the parameters of a connecting rod. Based on the proposed method, a novel measuring head is developed by using several LDSs. The measurement model and key measuring factors are discussed in detail. The experiment results show that the measuring error of dimensional parameters is within 10 μm, while the measuring error of the positional parameter is within 15 μm. Hence, the method proposed is proved to have acceptable accuracy and reliability. It provides an effective way for in-situ quality control of the dimensional and positional parameters.

## Figures and Tables

**Table 1:** The intrinsic parameters.

**The Lower Bore Gauge (°, mm)**					
*θ*_1_	*θ*_2_	*θ*_3_	*θ*_4_	*θ*_5_	*r*
29.832	29.316	1.283	−1.119	1.938	71.3320
**The Upper Bore Gauge (°, mm)**					
*θ*_1_	*θ*_2_	*θ*_3_	*θ*_4_	*θ*_5_	*r*
29.727	29.576	−0.345	1.461	1.618	70.9794

**Table 2:** The critical parameters of the measured connecting rod.

	Inner Diameter of Smaller Hole	Inner Diameter of Bigger Hole	Hole–Center Distance
Measured value (mm)	150.0172	275.0231	350.0063
Standard value (mm)	150.0227	275.0292	350.0185
Measuring error (μm)	5.5	6.1	12.2
